# Navigating the Fitness Landscape: Host Density, Epistasis, and Clonal Interference Drive Divergent Evolutionary Pathways in Phage Qβ

**DOI:** 10.3390/ijms26189020

**Published:** 2025-09-16

**Authors:** Mara Laguna-Castro, Pilar Somovilla, Víctor López-Muñoz, Luis F. Pacios, Ester Lázaro

**Affiliations:** 1 Centro de Astrobiología (CAB), CSIC-INTA, Carretera de Ajalvir Km 4, 28850 Torrejón de Ardoz, Madrid, Spain; mlaguna@cab.inta-csic.es (M.L.-C.); psomovilla@cab.inta-csic.es (P.S.); victorlm1801@gmail.com (V.L.-M.); 2 Departamento de Biotecnología-Biología Vegetal, Universidad Politécnica de Madrid (UPM), 28040 Madrid, Spain; luis.fpacios@upm.es

**Keywords:** bacteriophage Qβ, experimental evolution, fitness trade-offs, epistasis, clonal interference, host density, fitness landscape, evolutionary pathways

## Abstract

Understanding how ecological factors shape viral evolution is essential for predicting adaptation in RNA viruses. In this study, we investigated the evolutionary dynamics of bacteriophage Qβ under varying host densities, focusing on two nonsynonymous mutations—A1930G and C2011A—located in the A1 protein. Using experimental evolution, phenotypic assays, and competition experiments, we found that C2011A is consistently selected at low bacterial densities, enhancing viral entry but reducing burst size. In contrast, A1930G is fixed at high densities, despite similar phenotypic effects, suggesting its advantage arises from interactions with additional mutations. Clonal analysis revealed that compensatory or beneficial mutations modulate the fitness of A1930G, enabling its fixation. The absence of both mutations in the same genome points to negative epistasis, confirmed by the poor performance of the double mutant generated by site-directed mutagenesis. Sequencing of intermediate transfers showed early emergence of A1930G, but its fixation was prevented by clonal interference with C2011A. These findings highlight how host availability, fitness trade-offs, epistasis, and competition among variants shape the adaptive landscape of RNA viruses.

## 1. Introduction

Lytic bacteriophages follow a life cycle consisting of an extracellular phase and an intracellular phase. During the extracellular phase, phage particles are exposed to environmental stressors such as temperature fluctuations, ultraviolet radiation, and interactions with various substrates, all of which can compromise their infectivity. The intracellular phase begins when a phage successfully adsorbs to a susceptible bacterial host by binding to specific surface receptors. This is followed by the penetration of the viral genome into the host cytoplasm, replication of viral components, assembly of new virions, and ultimately host cell lysis and release of progeny.

Three key parameters define the efficiency of the lytic cycle: the latent period, which is the time between genome entry and the onset of progeny release; the burst size, which refers to the number of phages released per infected cell; and the adsorption rate constant (*k*), which reflects the probability of successful adsorption per unit time and per host cell [1,2,3,4]. This constant depends on viral and host properties, as well as environmental conditions such as temperature and medium viscosity [5]. In addition, the efficiency of phage entry into bacteria also depends on the concentrations of both phages and hosts, although due to mass action kinetics, host concentration is generally considered to exert a greater influence [6].

The lytic cycle parameters are subject to evolutionary pressures and can be modulated by natural selection to increase viral fitness under different ecological conditions [7,8,9,10,11]. However, optimization of a particular trait often occurs at the cost of others, a phenomenon known as fitness trade-offs [6,8,12,13]. For instance, a longer latent period may increase burst size but delay the onset of new infections [14,15,16]. In this line, experimental evolution studies have shown that under high host density, phages tend to evolve shorter latent periods to maximize the number of infection cycles per unit time [17,18]. Conversely, under low host density, longer latent periods may be favored to avoid rapid depletion of available hosts and allow time for host population recovery. Similarly, increased extracellular stability may come at the cost of reduced intracellular replication efficiency [19,20,21,22,23], although this trade-off is not universally observed [24,25,26].

In previous experimental evolution studies conducted in our laboratory using the RNA bacteriophage Qβ (family *Fiersviridae*, genus *Qubevirus*) [27], we serially propagated viral populations in *Escherichia coli* under varying host densities [28]. We found that at low bacterial densities (≤3 × 10^7^ cfu/mL), the mutation C2011A was consistently selected. This mutation enhanced infectivity by facilitating viral entry into host cells, albeit at the cost of reducing the burst size. This pattern resembles a classic trade-off between transmission (favored by increased entry efficiency) and virulence (favored by increased replication efficiency) commonly observed in many human viruses [29,30,31,32,33]. In contrast, at high bacterial densities (3 × 10^8^ cfu/mL), this mutation was never selected and was consistently outcompeted by the wild-type virus. These results suggest that when the probability of encountering a host is high, maximizing burst size becomes more advantageous than enhancing entry efficiency.

Interestingly, under high-density conditions, we observed the consistent fixation of a different mutation, A1930G, which was absent from all low-density evolved lines. This mutation has frequently emerged in evolution experiments conducted in our lab, suggesting it confers a selective advantage [26,34,35,36]. However, its absence under low host densities point to that its benefit may be modulated by ecological conditions—specifically, host availability—and may be antagonistic to that of C2011A. Our observations could be well explained if A1930G either reduce or maintain entry efficiency while enhancing burst size, making it advantageous in high-density environments but disadvantageous when host availability is limited.

Qβ infects *E. coli* by binding to the F conjugative pilus. It has a 4217-nucleotide positive-sense single-stranded RNA genome encoding four proteins: the multifunctional A2 protein (involved in receptor binding, lysis, and maturation) [37,38], the major coat protein (CP), the A1 protein (produced via stop codon readthrough of the CP protein gene and present in low copy number in the capsid) [39], and the RNA-dependent RNA polymerase (replicase). The A1 protein consists of two domains: the CP domain and the readthrough domain, which projects outward from the capsid surface. Like other RNA viruses, Qβ replicates with high error rates [40], generating highly heterogeneous populations in which most single and many double mutants can arise within a few generations [41]. These variants are then subject to natural selection, with only a subset reaching appreciable frequencies [42]. The simultaneous presence of multiple beneficial mutations in the same population can lead to clonal interference, a phenomenon in which competing lineages hinder each other’s fixation, potentially shaping the evolutionary trajectory [43,44,45,46,47,48].

Both the A1930G and C2011A mutations are located in the readthrough domain of the A1 protein (resulting in amino acid substitutions Q195R and T222N), whose function remains poorly understood but is essential for infection [39]. The fact that C2011A enhances viral entry suggests that A1 may facilitate pilus localization. Another possibility is that A1 interacts with A2 to orient the virion correctly for genome delivery. In addition to the possibility of antagonistic effects between A1930G and C2011A, their mutually exclusive selection, together with their location in the same protein, raises the possibility of negative epistasis, where the presence of one mutation interferes with the benefit of the other [49,50,51,52].

In this study, we aim to explore two non-mutually exclusive hypotheses: (i) that A1930G and C2011A confer density-dependent fitness advantages by modulating distinct infection parameters and (ii) that negative epistatic interactions and/or clonal interference prevent their co-selection under low host density. By dissecting the phenotypic effects and potential interactions of these mutations, we seek to better understand how ecological context shapes the adaptive landscape of RNA viruses.

## 2. Results

### 2.1. Parameters of the Infection Cycle of a Single Mutant Containing Mutation A1930G

**Figure 1:**
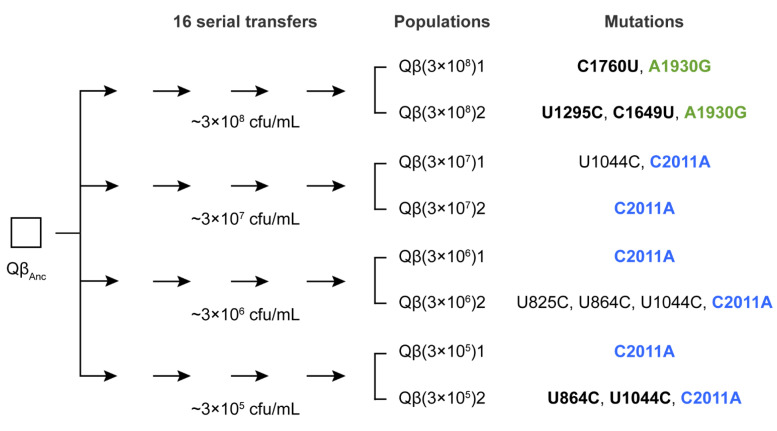
Scheme of the evolution experiment on which this study is based [28]. The ancestral virus Qβ_Anc_ (obtained from a cDNA clone; see Section 4.1) was propagated for 16 serial transfers at the bacterial densities indicated. Evolutionary lineages obtained at transfer number 16 were denoted Qβ(3 × 10^8^), Qβ(3 × 10^7^), Qβ(3 × 10^6^), and Qβ(3 × 10^5^)_,_ indicating the bacterial density (expressed in cfu/mL and shown in brackets). Two replicates were performed for each condition. The mutations that appear in the consensus sequence of each line are shown on the right. A1930G and C2011A are highlighted in green and blue, respectively. Fixed mutations are shown in bold.

In a previous evolution experiment [28] using bacteriophage Qβ propagated through 16 serial transfers at varying bacterial densities, mutation A1930G was exclusively selected in the lines evolved at the highest bacterial density (3 × 10^8^ cfu/mL). In that study, the ancestral virus Qβ_Anc_ was a wild-type clone derived from a cDNA copy of Qβ, as described in Section 4. In contrast, mutation C2011A was consistently selected at all lower densities that supported viral persistence throughout the experiment (Figure 1). This pattern suggests that the selective advantage of these mutations may depend on host availability. If this hypothesis is correct, the mutations should differentially affect key parameters of the Qβ infection cycle. While these parameters have already been characterized for a single mutant carrying mutation C2011A (Qβ_C2011A_) [28], we sought to compare them with those obtained for another mutant carrying only mutation A1930G (Qβ_A1930G_), which was available in our laboratory [26].

**Figure 2:**
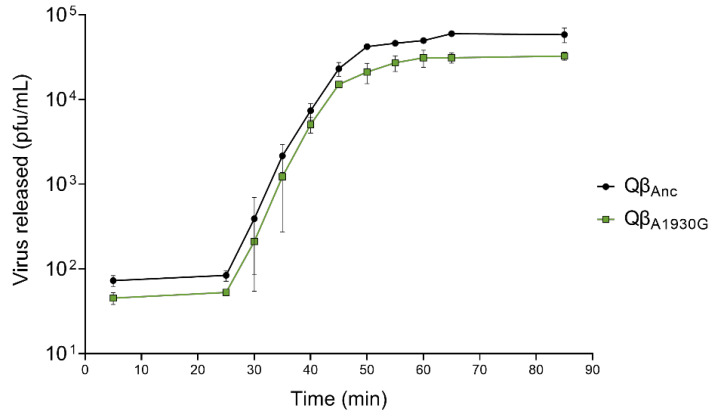
One-step growth curves obtained for Qβ_Anc_ and Qβ_A1930G_. Experimental details are described in Section 4.

To determine the latent period of Qβ_A1930G_ relative to the wild-type virus (represented by Qβ_Anc_, as indicated in Section 4), we performed one-step growth curves (Figure 2). Both viruses exhibited a latent period of 25 min, which is comparable to the 30-min latent period previously reported for Qβ_C2011A_ and Qβ_Anc_ [28]. It is important to note that virus–host contact was limited to five minutes in this experiment to minimize differences in adsorption efficiency. Minor differences between assays may also arise from variations in bacterial physiological state, culture media batches, or subtle, hard-to-control laboratory conditions.

The burst size of Qβ_A1930G_ was estimated at 378 ± 65 pfu per infected cell, significantly lower than that of Qβ_Anc_ (763 ± 155 pfu) (*p* < 0.05, two-tailed Welch test) but similar to that of Qβ_C2011A_ (411 ± 27 pfu; *p* > 0.05, two-tailed Welch test).

**Figure 3:**
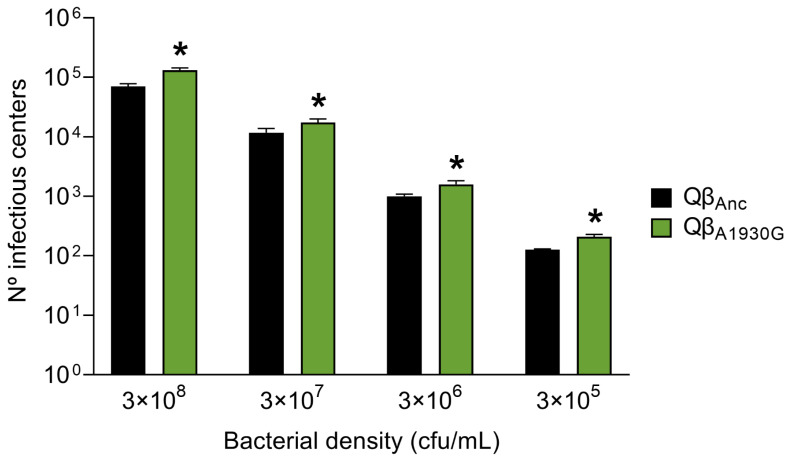
Number of infectious centers produced by Qβ_Anc_ and Qβ_A1930G._ A total of 10^5^ pfu of either Qβ_Anc_ (black bars) or Qβ_A1930G_ (green bars) were incubated for 10 min with the bacteria concentrations indicated below the figure (3 × 10^8^, 3 × 10^7^, 3 × 10^6^, and 3 × 10^5^ cfu/mL). After this time, the samples were processed as indicated in Section 4.2 of Section 4. Each bar represents the average of three determinations. Asterisks indicate statistically significant differences from Qβ_Anc_ (*p* < 0.05, Welch test with Benjamini–Hochberg correction for multiple comparisons).

To assess viral entry efficiency, we determined the number of infectious centers produced by Qβ_Anc_ and Qβ_A1930G_ after a 10-min incubation period with different bacterial concentrations and further processing of the samples as described in Section 4.2. As shown in Figure 3, the mutant exhibited significantly enhanced entry efficiency relative to the wild-type virus (*p* < 0.05; two-tailed Welch test with Benjamini–Hochberg correction). However, the differences between the entry efficiency of Qβ_A1930G_ and Qβ_C2011A_ (determined in our previous study) [28] were minimal. The mutant Qβ_A1930G_ increased the number of infectious centers relative to the ancestral virus Qβ_Anc_ by 1.9-, 1.5-, 1.6-, and 1.7-fold at host cell densities of 3 × 10^8^, 3 × 10^7^, 3 × 10^6^, and 3 × 10^5^ cfu/mL, respectively. In comparison, the mutant Qβ_C2011A_ showed increases of 1.7-, 2.7-, 1.8-, and 1.3-fold under the same conditions. We did not perform a kinetic analysis for Qβ_A1930G_. Nevertheless, since the constant of formation of infectious centers was determined for Qβ_C2011A_ in our previous study [28] and both mutants exhibited comparable entry profiles in the endpoint measurements after 10 min, we assumed that the adsorption constant for Qβ_A1930G_ is likely similar.

In summary, Qβ_A1930G_ and Qβ_C2011A_ displayed broadly similar infection cycle parameters. While subtle differences cannot be excluded, they were not evident under the conditions tested in this study. Thus, the selective advantages of each mutant probably depend on factors such as ecological conditions, genomic context, and the presence of other competitor mutants in the population.

### 2.2. Selective Value of Mutation A1930G

**Figure 4:**
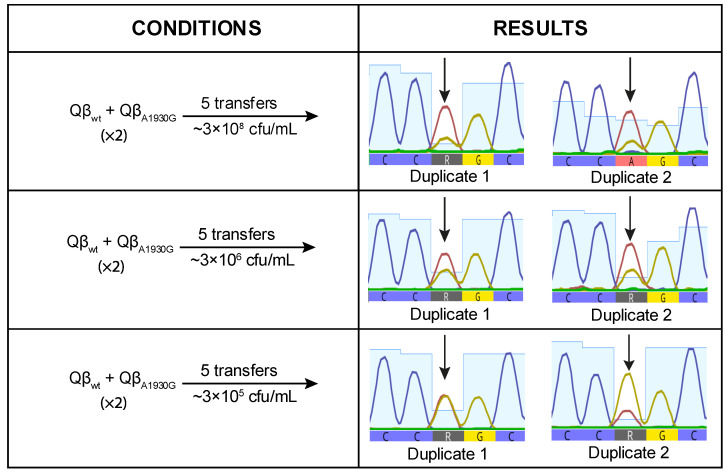
Competition experiment between Qβ_Anc_ and Qβ_A1930G._ The left panel shows the experimental conditions (see Section 4 for details). The right panel displays chromatograms of the populations obtained after the competition, centered around nucleotide position 1930 (indicated by an arrow). At this position, the red peak represents the wild-type nucleotide (A) and the ochre peak the mutant nucleotide (G). When both nucleotides are present, this is indicated by the letter R.

To investigate whether, despite having a burst size value lower than that of Qβ_Anc_, Qβ_A1930G_ has a selective advantage on its own, we performed a competition experiment between both viruses. Equal amounts of each virus were used to initiate the infection, and the resulting progeny was propagated through five serial transfers. The experiment was conducted in duplicate at three bacterial concentrations: 3 × 10^8^ cfu/mL, 3 × 10^6^ cfu/mL, and 3 × 10^5^ cfu/mL. At the final transfer, we analyzed position 1930 of the viral genome to determine whether the mutation had become dominant or not (Figure 4).

When the virus mixture was propagated at high bacterial concentration (3 × 10^8^ cfu/mL), mutation A1930G was detected at very low frequency, suggesting a deleterious effect under this condition. This result was unexpected, as A1930G had previously become fixed in lines evolved at this density (Figure 1). At lower bacterial densities, the negative effect appeared to be mitigated, as indicated by increased signal intensity for the mutant nucleotide (Figure 4). However, the mutant peak exceeded the wild-type peak in only one replicate at the lowest bacterial density tested (3 × 10^5^ cfu/mL). These findings suggest that A1930G does not confer a selective advantage on its own at high density and may require a favorable mutational background or compensatory mutations to be maintained.

**Figure 5:**
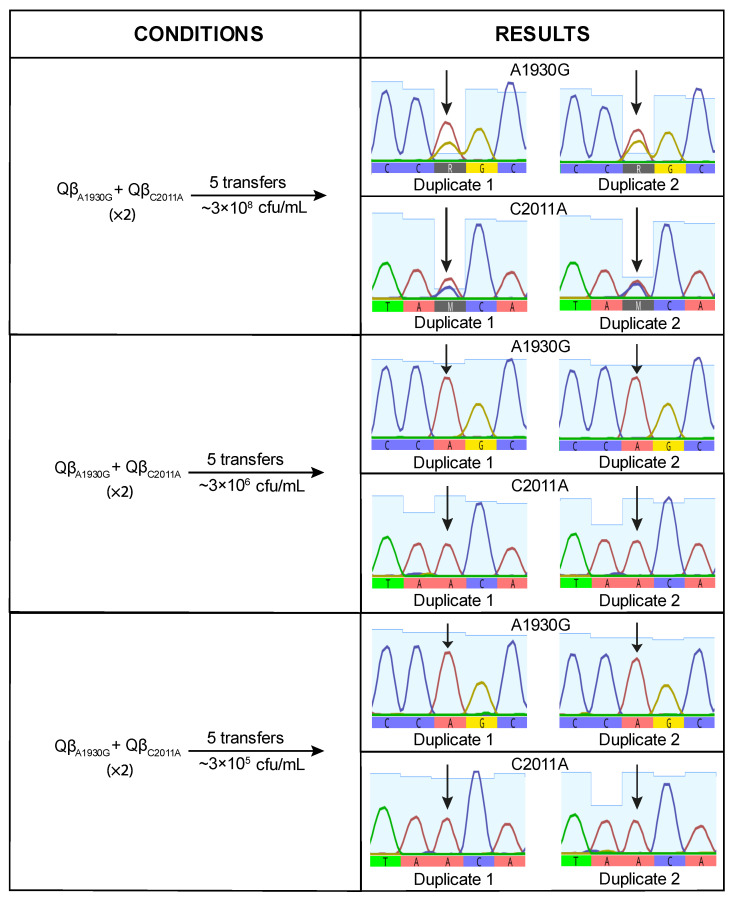
Competition experiment between Qβ_A1930G_ and Qβ_C2011A._ The left panel shows the experimental conditions (see Section 4 for details). The right panel displays chromatograms of the populations obtained after the competition, centered around nucleotide positions 1930 and 2011 (indicated by arrows). At position 1930, the red peak represents the wild-type nucleotide (A) and the ochre peak the mutant nucleotide (G). When both nucleotides are present, this is indicated by the letter R. At position 2011, the blue peak represents the wild-type nucleotide (C) and the red peak the mutant nucleotide (A). The letter M represents a mixture of C and A.

We also performed competition experiments between Qβ_A1930G_ and Qβ_C2011A_. At high bacterial density (3 × 10^8^ cfu/mL), both mutants coexisted. However, at lower densities (3 × 10^6^ and 3 × 10^5^ cfu/mL), Qβ_A1930G_ was completely outcompeted (Figure 5). These results indicate that, in the wild-type genomic background, C2011A confers a greater selective advantage than A1930G under low host density conditions.

### 2.3. Selective Value of Mutation A1930G in Different Mutational Contexts

To investigate the selective value of mutation A1930G in different mutational contexts, we isolated biological clones from each of the two evolutionary lines propagated at high bacterial density in this experiment, shown in Figure 1 [28]. These clones were then competed against the virus Qβ_Anc_ at two bacterial concentrations (3 × 10^8^ and 3 × 10^6^ cfu/mL) or against the virus Qβ_C2011A_ (in this case, only at low bacterial density).

In addition to mutation A1930G, the consensus sequence of the line Qβ(3 × 10^8^)1 contained C1760U (synonymous), while line Qβ(3 × 10^8^)2 carried mutations U1295C (resulting in change F411S in the A1 protein) and C1649U (synonymous). Biological clones isolated from each line were sequenced to select those that could provide the most insight into how the selective value of mutation A1930G is influenced by the presence of additional mutations. The selected clones and the results of the competition experiments are summarized in Table 1.

Clones selected from line Qβ(3 × 10^8^)1 (clones 1.1 and 1.2) carried mutation A1930G within two distinct mutational contexts. Clone 1.1 contained mutation C1760U, making its sequence identical to the consensus of the population from which it was isolated. This clone completely displaced both Qβ_Anc_ and Qβ_C2011A_ viruses at the tested bacterial densities. The result suggests that mutation C1760U, despite being synonymous, may compensate for the deleterious effects of mutation A1930G. The combination of both mutations provides an advantage even greater than that conferred by C2011A under conditions in which the latter is typically favored (3 × 10^6^ cfu/mL). Clone 1.2 carried A1930G along with U168C (synonymous) and C2223A (resulting in change V293I in the A1 protein). Although mutation C1760U was fixed in the population Qβ(3 × 10^8^)1, it was absent in this clone. Clone 1.2 also fully displaced Qβ_Anc_ at both high and low bacterial densities. However, it was unable to completely displace the virus Qβ_C2011A_, indicating that this combination of mutations is less advantageous than that of clone 1.1 under low-density conditions.

Clones selected from line Qβ(3 × 10^8^)2 (clones 2.1 and 2.2) carried the mutations present in the consensus sequence of the population (U1295C, U1649C, and A1930G), along with additional mutations. Clone 2.1 included mutation A3910C (resulting in change E519A in the replicase), while clone 2.2 carried mutation U2847C (resulting in change Y165H, also in the replicase). Both clones were capable of displacing the virus Qβ_Anc_ at both high and low bacterial densities. However, neither clone was able to displace Qβ_C2011A_ at low bacterial density. After five competition transfers, mutation C2011A tended to increase in frequency when co-propagated with clone 2.1 but decreased when co-propagated with clone 2.2.

### 2.4. Analysis of the Early Presence of Mutation A1930G During the Evolution of Qβ

Since the negative effects of mutation A1930G can be mitigated—both at high and low bacterial densities—by the presence of additional mutations, it is conceivable that it could become fixed in populations evolving under low-density conditions as it does at high density. However, the fact that A1930G was not fixed in any of the six lines evolved at low bacterial density suggests alternative explanations. One possibility is that mutation C2011A outcompeted A1930G before the mutations that could mitigate its negative effect could emerge. To explore this hypothesis, we analyzed the consensus sequence (from nucleotide 50 to 2100, covering A1930G, C2011A, and potential compensatory sites) of all evolutionary lines labeled as “1” in Figure 1 at several transfers previous to transfer 16 (see Supplementary Material, Table S1).

At transfer 8, mutation A1930G was detected as a low-frequency polymorphism, either alone (in the line propagated at 3 × 10^8^ cfu/mL) or coexisting with C2011A (in lines propagated at lower bacterial densities). In Qβ(3 × 10^8^)1, mutation C1760U became detectable at transfer 10, after which both A1930G and C1760U increased in frequency until fixation. In contrast, C1760U was absent in all sequenced transfers of lines evolved at lower densities. In these lines, clear signs of competition between A1930G and C2011A were observed: as the transfer series progressed, C2011A increased in frequency until fixation, while A1930G declined and eventually became undetectable.

These results suggest that mutation A1930G arises early during Qβ evolution, regardless of bacterial density. Its eventual fixation depends on its selective value under specific propagation conditions, the presence of accompanying mutations, and potential competition with other adaptive mutations such as C2011A.

### 2.5. Analysis of the Selective Value of the Combination of Mutations A1930G and C2011A

The absence of the simultaneous fixation of mutations A1930G and C2011A in any of the evolutionary lines propagated at low bacterial density suggests that their combined presence does not confer an advantage beyond that provided by the most beneficial individual mutation (C2011A). An alternative explanation is that these mutations interact negatively, reducing the overall fitness of the double mutant compared to each single mutant alone (negative epistasis).

To evaluate the combined effect of both mutations within the same genome, we constructed a double mutant that contains the two mutations in the genomic context of the wild-type virus. The double mutant was obtained using Qβ_A1930G_ as the template and introducing mutation C2011A by site-directed mutagenesis. The same primers and protocol previously described for the construction of the single C2011A mutant were used [28]. Duplicate competition experiments were then performed between the double mutant and the viruses Qβ_Anc_, Qβ_A1930G_, and Qβ_C2011A_. At high bacterial concentrations (3 × 10^8^ cfu/mL), the double mutant was consistently outcompeted by all competitors. However, at lower bacterial densities (3 × 10^6^ cfu/mL), the double mutant successfully displaced both Qβ_Anc_ and Qβ_A1930G_. In contrast, Qβ_C2011A_ consistently outcompeted the double mutant in both replicates.

These results suggest that mutation C2011A plays a dominant role in determining the fitness of the double mutant. At high bacterial density—where C2011A is disadvantageous—the double mutant is eliminated. At low bacterial density—where C2011A confers a selective advantage—the double mutant performs better than Qβ_Anc_ and Qβ_A1930G_, but not better than Qβ_C2011A_ alone. This indicates that the presence of A1930G may partially offset the beneficial effects of C2011A under these conditions.

To further quantify the interaction between mutations A1930G and C2011A, we calculated the relative replicative fitness of each single mutant and the double mutant under two bacterial density conditions. Relative replicative fitness was defined as the ratio between the viral titers obtained for each mutant and those obtained for the ancestral virus (Qβ_Anc_) in the same replication assay (see Section 4.5 in Section 4). At high bacterial density (3 × 10^8^ cfu/mL), the fitness values of Qβ_A1930G_ and Qβ_C2011A_ were 0.91 and 1.56, respectively, predicting an additive relative fitness of approximately 1.42 for the double mutant. However, the observed value was 0.07, indicating strong negative epistasis. At low bacterial density (3 × 10^6^ cfu/mL), the expected additive value was 2.22 (based on single mutant fitness values of 0.63 and 3.52 for Qβ_A1930G_ and Qβ_C2011A_, respectively), while the observed value was 0.20. These findings confirm that the two mutations interact negatively, reducing overall fitness when combined.

To investigate the mechanistic basis of this reduced fitness, we compared key infection parameters among the three viruses. Entry efficiency assays carried out at a bacterial density of 3 × 10^7^ cfu/mL revealed no significant differences between the double mutant and either single mutant. However, the burst size of the double mutant was lower (283 ± 81 pfu per infected cell) than that of Qβ_C2011A_ or Qβ_A1930G_. Although the difference was not significant (*p* > 0.05, two-tailed Welch test), it suggests that the reduced replication output may underlie the lower competitive performance of the double mutant.

### 2.6. Structure of the Qβ A1 Protein

To obtain a deeper insight in the mechanisms underlying the effects of mutations A1930G and C2011A either alone or combined in the same genome, we predicted model structures of the A1 protein using AlphaFold 3. The results show nearly indistinguishable coat protein (CP) domains (residues 1–132) and similar A1 domains (residues 133–328), although the linker segment 133–150 exhibits different spatial orientations due to its disorder (Supplementary Material Figure S1). The CP domain in the models is virtually identical to the domain shown by this protein in the cryo-EM structure of Qβ capsid (PDB ID 7LHD) whereas the A1 domain is structurally similar to the X-ray structure of the read-through domain of A1 (PDB ID 3RLK). In this latter domain, the secondary structure with irregular long loops in the models is well conserved with respect to the X-ray structure. Both mutant positions are in the middle of loops, being position 222, closer to the CP domain, and position 195, farther from the CP domain and oriented outwards (Figure S1).

## 3. Discussion

Understanding how viruses adapt to changing ecological conditions is central to evolutionary virology. RNA viruses, in particular, are characterized by high mutation rates, large population sizes, and short generation times, which together generate highly dynamic and heterogeneous populations capable of rapid adaptation to new selective pressures [41,42]. In this study, we investigated how two specific mutations in the A1 protein gene—A1930G (Q195R) and C2011A (T222N)—are differentially selected in bacteriophage Qβ depending on host density. Our findings reveal a complex interplay between ecological conditions, mutational context, fitness trade-offs, and competition among different mutants, highlighting the multifactorial nature of adaptation in RNA viruses.

Numerous studies have shown that host density is a key ecological parameter influencing viral evolution [11,28,53,54,55,56]. In the case of bacteriophage Qβ, mutation C2011A was consistently selected in populations evolved under low bacterial densities (≤3 × 10^7^ cfu/mL), where it enhanced viral entry [28]. In contrast, this mutation was never detected in virus lines evolved at high bacterial density (3 × 10^8^ cfu/mL). Determination of the infection cycle parameters for a mutant carrying only C2011A revealed that the increased entry efficiency came at the cost of a reduced burst size, illustrating a classic trade-off between transmission and virulence (represented here by replication efficiency). Each transfer in our evolution experiment lasted two hours, a period during which two to three infection cycles are possible if sufficient viruses and bacteria are present. Therefore, when hosts are abundant, increasing the rate of virus entry offers no advantage—especially if it reduces the number of progeny viruses capable of initiating new infections. However, under conditions of host scarcity, the relative importance of these parameters is reversed, and enhancing entry efficiency becomes advantageous, even at the cost of reduced burst size.

In contrast, mutation A1930G was fixed exclusively in populations evolved at high bacterial density. This mutation has also been frequently observed in previous evolutionary experiments conducted in our laboratory, suggesting a generally positive effect [26,34,35,36]. This makes its absence in the lines evolved at low bacterial density particularly intriguing. Initially, we hypothesized that the infection cycle parameters of viruses carrying A1930G might differ from those of viruses with C2011A. However, our measurements showed that A1930G also increased viral entry and reduced burst size in a manner similar to C2011A. These results raise the question of why A1930G becomes fixed under high bacterial density, where the cost in burst size should be detrimental, as observed with C2011A. Competition experiments between Qβ_Anc_ and Qβ_A1930G_ indicated that, in the genomic context of the wild-type virus, A1930G has a deleterious effect that diminishes as bacterial density decreases. However, under low-density conditions, C2011A consistently outcompeted A1930G. This apparent paradox suggests that the fixation of A1930G at high bacterial density is not due to its individual effect but rather to its interaction with other mutations—either through compensatory evolution or hitchhiking with beneficial variants.

Clonal analysis of lines evolved at high bacterial density revealed that A1930G was accompanied by other mutations, including synonymous and nonsynonymous substitutions in the A1 protein and the replicase. Some of these combinations conferred a competitive advantage over the wild-type virus and, in some cases, over C2011A. These findings suggest that compensatory or synergistic interactions can modulate the fitness effects of A1930G, enabling its persistence and eventual fixation under specific conditions. Particularly noteworthy is the combination of A1930G with C1760U. The latter is a synonymous mutation, so its effect likely occurs at the level of RNA structure, potentially facilitating virus assembly or more efficient genome replication.

The complete absence of A1930G—even as a polymorphism—in the final transfer of any lines evolved at low bacterial density is especially surprising given that A1930G is a transition mutation and thus expected to arise more frequently than the transversion C2011A. One possible explanation is that, although both mutations enhance Qβ entry efficiency, they may do so via different mechanisms with distinct effects depending on the multiplicity of infection (moi). In our evolution experiment (Figure 1), the viral input was kept constant (10^7^ pfu), implying that the moi was <1 at high bacterial density and ≥1 at low density. Intriguingly, C2011A was fixed at moi ≥ 1, whereas A1930G did so only when the moi was <1. To disentangle the effects of moi from those of bacterial density, we performed an additional evolution experiment in which Qβ was propagated at high bacterial density using a viral input that results in an moi of approximately 10—matching the higher moi values used in low-density conditions. Under this condition, mutation C2011A was not selected, suggesting that moi alone does not explain its fixation. We are currently conducting further additional evolution experiments to test the opposite scenario—whether A1930G can be selected at low host density and low moi. These experiments involve a wide range of combinations of host densities and viral population sizes and will be the subject of a future study.

It has been reported that Qβ genome penetration triggers detachment of the F pilus, [57]. If additional viruses are bound to the same pilus, they may be removed along with it, and it is unclear whether these viruses can be released or remain unavailable for future infections. In such a scenario, a mutation that increases adsorption to pili could be detrimental at high moi, potentially explaining why A1930G (Q195R) is selected only under low moi conditions. At low bacterial density, increased adsorption can become advantageous, as the low number of bacteria makes virus–host encounters less frequent, reducing the likelihood of multiple viruses binding to the same pilus. The results of our competition experiments showing that the negative effect of A1930G decreases with bacterial density (even if that means increasing the moi) are in agreement with this possibility.

In contrast, C2011A (T222N) may facilitate viral entry through a different mechanism that does not involve increased adsorption but rather improves other aspects of genome penetration or virus orientation relative to the pilus—mechanisms that have been well studied in phage MS2 [58]. If this is the case, C2011A would consistently outperform A1930G at low bacterial densities, particularly when viral input is high, as it happens in the evolution experiment shown in Figure 1. To obtain a deeper insight into the molecular mechanisms involved in Qβ adsorption and genome penetration, we are currently investigating potential interactions between A1 and A2 proteins in the wild-type virus and both mutants using AlphaFold and molecular dynamics simulations. Since the CP domain of A1 must replace one of the CP monomers of the external surface of the Qβ capsid, interaction of A1 with A2 or the F pilus should occur via the readthrough domain of A1. AlphaFold structural models suggest that position 222 would be closer to A2 than position 195, which is oriented outwards (Supplementary Figure S1). It can thus be conjectured that mutation T222N would influence the interaction with A2 whereas mutation Q195R would have some effect on the interaction with the F pilus. Being both residues located in the middle of long loops, they have conformational freedom to interact favorably with their targets in A2 and/or in the F pilus.

Our data also suggest that negative epistasis may prevent the co-selection of A1930G and C2011A. The double mutant constructed in this study failed to outperform the single C2011A mutant under any tested condition and was consistently outcompeted at high host density. This suggests that the combined presence of both mutations may interfere with optimal virus replication, limiting their joint fixation. The lower burst size of the double mutant relative to the single mutants supports this explanation. Such epistatic constraints are increasingly recognized as important factors shaping viral evolutionary trajectories, particularly in RNA viruses where multiple beneficial mutations can arise and interact within short timeframes.

Another key insight from this study is the role of clonal interference. Sequencing of intermediate transfers revealed that A1930G often emerged early during evolution, even under low host density. However, it was consistently outcompeted by C2011A in these conditions. This supports the idea that the timing of mutation appearance, combined with relative fitness advantages, determines which variants ultimately fix in the population. Clonal interference—where multiple beneficial mutations compete within a population—is a hallmark of large viral populations when recombination is absent and can delay or prevent the fixation of otherwise advantageous mutations [43,44,45,46,47,48].

We also examined a previously published multiple sequence alignment of the A1 protein from different alloleviviruses [59]. The alignment shows that the positions corresponding to Q195 and T222 are not strictly conserved, suggesting that these residues may contribute to species-specific functional adaptations. This observation supports the hypothesis that the mutations studied here affect interactions unique to Qβ, potentially explaining their context-dependent selection.

Overall, our findings underscore the context-dependent nature of viral adaptation and the role of both ecological and genetic interactions in shaping evolutionary trajectories. The mutually exclusive selection of A1930G and C2011A across host densities, their phenotypic effects, and their negative interaction when combined illustrate the complexity of the adaptive landscape in RNA viruses. These insights contribute to a deeper understanding of how fitness trade-offs, epistasis, and clonal interference influence viral evolution and may inform strategies for predicting evolutionary outcomes in rapidly evolving pathogens, including pathogenic RNA viruses, where similar principles govern the emergence of drug resistance, immune escape, and host adaptation.

Future work should aim to dissect the molecular mechanisms by which these mutations affect viral fitness—particularly their impact on receptor interaction and genome replication. Additionally, integrating mathematical modeling with experimental evolution under a broader range of conditions (e.g., bacterial density and viral population size) could help predict the circumstances under which specific mutations are likely to fix and how viral populations navigate complex fitness landscapes over time.

## 4. Materials and Methods

### 4.1. Virus and Bacteria: Standard Procedures for Infection

The plasmid pBRT7Qβ, which carries a cDNA copy of bacteriophage Qβ cloned into the pBR322 vector [60,61], was used to transform *Escherichia coli* DH5-α. This strain supports viral expression but is non-permissive to infection due to the absence of the Qβ receptor. The supernatant from an overnight culture of a transformed colony was used to infect *E. coli* Hfr (Hayes strain) [62] in semisolid agar. A lytic plaque was randomly selected, and the virus progeny was isolated. A total of 10^6^ plaque-forming units (pfu) from this isolate were used to infect an *E. coli* Hfr culture under standard conditions (described below). The resulting viral supernatant, referred to as Qβ_Anc_, served as the ancestral virus for all evolutionary lineages propagated under different bacterial densities (see Figure 1). The consensus sequence of Qβ_Anc_ showed no mutations relative to the Qβ cDNA cloned in pBR322 and was therefore considered equivalent to the wild-type virus.

Infections in liquid medium were performed in 12 mL tubes containing 0.5 mL of bacterial suspension, the desired number of pfu in 100 µL of phage buffer (PB: 1 g/l gelatin, 0.05 M Tris-HCl, pH 7.5, and 0.01 M MgCl_2_), and 0.4 mL of nutrient broth (NB: 8 g/L Nutrient Broth, Merck, and 5 g/L NaCl). Bacteria were freshly prepared by growing *E. coli* to an OD_600_ of 0.8, which corresponds to approximately 6 × 10^8^ cfu/mL, based on standard estimations (Agilent OD calculator, Santa Clara, CA, USA). When necessary, cultures were serially diluted in NB to achieve lower bacterial concentrations. Infections were incubated at 37 °C for 2 h with vigorous shaking (250 rpm). Viral supernatants were collected by centrifugation at 13,000× *g* and stored at 4 °C for short-term use (up to 15 days) or at −80 °C for long-term storage. Viral titers were determined by plaque assay and expressed as pfu/mL.

### 4.2. Determination of Virus Entry into Bacteria

Triplicate 1 mL liquid cultures were prepared containing *E. coli* at four different densities (3 × 10^8^, 3 × 10^7^, 3 × 10^6^, and 3 × 10^5^ cfu/mL) and 10^5^ pfu of Qβ. Cultures were incubated at 37 °C with gentle shaking (75 rpm). After 10 min, 100 µL of 100 mM EDTA were added to each tube to halt viral entry. Control experiments confirmed that this EDTA concentration completely inhibited Qβ entry into *E. coli*, consistent with previous findings for other bacteriophages [63].

Following EDTA treatment, cultures were centrifuged at 13,000× *g* for 10 min at 4 °C. The supernatant was discarded, and the pellet was washed once with 500 µL of cold PB containing 10 mM EDTA. Pellets were then resuspended in PB without EDTA. The number of infectious centers was determined by plaque assay.

Parallel control cultures were incubated at 4 °C in the presence of 10 mM EDTA to estimate background levels of virus adsorption.

### 4.3. One Step Growth Curve

Duplicate 1 mL liquid cultures containing 3 × 10^8^ *E. coli* cells and 10^6^ pfu of either Qβ_Anc_ or Qβ_A1930G_ were incubated for 5 min at 37 °C in NB with gentle shaking (75 rpm). To halt further viral entry, cultures were diluted 10,000-fold in pre-warmed NB. The diluted cultures were then incubated at 37 °C in a static water bath and gently mixed by inversion only immediately before sampling, to minimize additional virus–host encounters.

At various time points, 250 µL aliquots were collected and centrifuged to separate the supernatant, which was then titrated by plaque assay to quantify released virus particles. The duration of the latent period was estimated as the intersection point between two regression lines: one fitted to the natural logarithm of virus titers during the exponential rise phase and the other to the pre-rise phase.

### 4.4. Burst Size Determination

Triplicate 1 mL liquid cultures containing 3 × 10^8^ *E. coli* cells and 10^5^ pfu of the indicated virus were incubated for 10 min at 37 °C in nutrient broth (NB) with gentle shaking (75 rpm) to allow viral entry. After this period, a 0.3 mL aliquot was taken to determine the number of infectious centers, as described above.

The remaining 0.7 mL of each culture was diluted 100,000-fold in pre-warmed NB and incubated at 37 °C with vigorous shaking (250 rpm) for 55 min to allow completion of the infection cycle. After incubation, 0.3 mL aliquots were collected and centrifuged to obtain the viral supernatant, which was titrated by plaque assay to quantify the number of extracellular viruses.

The burst size was calculated by dividing the number of pfu obtained after the incubation period (multiplied by the dilution factor) by the number of infectious centers measured prior to dilution.

### 4.5. Determination of Relative Replicative Fitness

The virus yield obtained in replication assays carried out in liquid medium was used as a measure of viral replicative ability. Triplicate 1 mL cultures containing the indicated bacterial density were inoculated with 10^4^ pfu of the virus under study (Qβ_Anc_, Qβ_A1930G,_ Qβ_C2011A_, and the double mutant containing both mutations). This inoculum size was chosen to avoid saturation of the replication system and ensure that differences in viral output reflected differences in replicative efficiency. After 2 h of incubation at 37 °C with shaking (250 rpm), viral supernatants were collected and titrated by plaque assay. Relative replicative fitness was defined as the ratio between the viral titer obtained for each mutant and that obtained for the ancestral virus Qβ_Anc_ in the same assay.

### 4.6. Competition Experiments

Competition assays were performed by co-infecting *E. coli* cultures at the specified bacterial density with equal amounts (10^6^ pfu) of each of the two competing viruses, in a final volume of 1 mL. After 2 h of incubation at 37 °C with shaking, the viral supernatant was collected, and 10^6^ pfu from the virus progeny were used to initiate the next transfer. Each competition was conducted in duplicate and continued for a total of five serial transfers.

To determine the outcome of the competition, the consensus sequences of the viral populations obtained after the fifth transfer were analyzed. The relative abundance of each competitor was inferred by inspecting the chromatograms at the nucleotide position that differentiated the two viruses.

### 4.7. RNA Extraction, cDNA Synthesis, PCR Amplification, and Nucleotide Sequencing

Viral RNA was prepared using QIAamp Viral RNA Mini kit (Qiagen, Hilden, Germany) to determine the consensus sequence either from biological clones or from complex virus populations. RNAs were used for cDNA synthesis with the avian myeloblastosis virus reverse transcriptase (Promega, Madison, WI, USA), followed by PCR amplification using Expand high-fidelity DNA polymerase (Roche Diagnostics, Mannheim, Germany). The pairs of oligonucleotide primers used for RT-PCR were the following: P1 forward (5′CTTTAGGGGGTCACCTCACAC3′) with P1 reverse (5′GGATGGGTCACAAGAACCGT3′) to amplify from nucleotide position 10 to 1595, P2 forward (5′GACGTGACATCCGGCTCAAA3″) with P2 reverse (5′CAACGGACGGAACATCTCCT3″) to amplify from nucleotide position 1109 to 2787, and P3 forward (5′GTGCCATACCGTTTGACT3′) with P3 reverse (5′GATCCCCCTCTCACTCGT3′) to amplify from nucleotide position 2254 to 4195. PCR products were column purified (Qiagen, Hilden, Germany) and subjected to standard Sanger sequencing using Big Dye Chemistry with an automated sequencer (Abi 3730 XL, Applied Biosystems, Perkin Elmer, Waltham, MA, USA). Sequences were assembled and aligned with Geneious Pro v4.8.5 (https://www.geneious.com). Mutations relative to the sequence of the virus Qβ_Anc_ were identified using the same software.

### 4.8. Structural Modeling of A1 Protein

The 3D structures of the wild-type form of A1 protein and its T222N, Q195R, and double T222N-Q195R mutants were obtained separately for the corresponding four sequences with AlphaFold 3 [64] (https://alphafoldserver.com/, accessed on 30 January 2025). Structural alignments of the four models superimposing their CP domain (residues 1–132) were computed with the CE method [65] implemented in PyMOL 3.1.6 [66], software also used to prepare molecular graphics.

## 5. Conclusions

C2011A is favored at low bacterial densities, enhancing viral entry but reducing burst size—a trade-off that is beneficial when hosts are scarce.

A1930G is selected at high bacterial densities, despite having similar phenotypic effects to C2011A. Its fixation appears to depend on interactions with other mutations, suggesting compensatory evolution or hitchhiking.

The absence of A1930G at low densities, despite being a more likely mutation (a transition), may be due to mechanistic differences in entry and moi-dependent effects.

The double mutant (A1930G + C2011A) shows negative epistasis, performing worse than either single mutant, which prevents their co-selection.

Clonal interference plays a key role: A1930G often arises early but is outcompeted by C2011A under low-density conditions.

## Figures and Tables

**Table 1:** Result of competition experiments between single mutants and clones isolated from populations Qβ(3 × 10^8^)1 and Qβ(3 × 10^8^)2.

Competition	Result	
	Host Density (cfu/mL)	
	3 × 10^8^	3 × 10^6^
**Clone 1.1 + Qβ_Anc_**	Clone 1.1	Clone 1.1
**Clone 1.1 + Qβ_C2011A_**	No tested	Clone 1.1
**Clone 1.2 + Qβ_Anc_**	Clone 1.2	Clone 1.2
**Clone 1.2 + Qβ_C2011A_**	No tested	Clone 1.2 = Qβ_C2011A_
**Clone 2.1 + Qβ_Anc_**	Clone 2.1	Clone 2.1
**Clone 2.1 + Qβ_C2011A_**	No tested	Clone 2.1 < Qβ_C2011A_
**Clone 2.2 + Qβ_Anc_**	Clone 2.2	Clone 2.2
**Clone 2.2 + Qβ_C2011A_**	No tested	Clone 2.2 > Qβ_C2011A_

## Data Availability

The data supporting the findings shown in this study are available on request to the corresponding author.

## Supplementary Materials

The following supporting information can be downloaded at: https://www.mdpi.com/article/10.3390/ijms26189020/s1.
