# Supplementary material for: Navigating the Fitness Landscape: Host Density, Epistasis, and Clonal Interference Drive Divergent Evolutionary Pathways in Phage Qβ
Source: Int J Mol Sci. 2025 Sep 16;26(18):9020. doi: 10.3390/ijms26189020 (PMC12469967; doi:10.3390/ijms26189020)
Supplement: Supplementary file 1 [file ijms-26-09020-s001.zip › Table S1.pdf]

Table S1. Frequency of the mutations observed in the consensus sequences of the evolutionary lines Qβ(3×10^8)1, Qβ(3×10^7)1, Qβ(3×10^6)1, and Qβ(3×10^5)1 at different transfers.

Number of Transfer	Mutation	Frequency of mutated nucleotide (%)			
		Evolutionary lines			
		3×10^8	3×10^7	3×10^6	3×10^5
8	C864T	0	0	8	0
	T1044C	0	0	0	0
	C1760T	0	0	0	0
	A1930G	6	26	23	9
	C2011A	0	27	46	62
10	C864T	0	0	0	0
	T1044C	0	0	0	0
	C1760T	13	0	0	0
	A1930G	18	30	22	10
	C2011A	0	52	70	81
12	C864T	0	0	0	0
	T1044C	0	0	0	0
	C1760T	22	0	0	0
	A1930G	56	21	13	0
	C2011A	0	74	85	100
16	C864T	0	7	8	0
	T1044C	0	17	6	0
	C1760T	61	0	0	0
	A1930G	100	0	0	0
	C2011A	0	100	100	100

The evolutionary lines are those depicted in Figure 1 of the main text. The genomic region analyzed corresponds to the segment spanning nucleotides 50 to 2100. The frequencies of the mutant nucleotides were estimated from chromatogram peak heights using the BioEdit visualization software. Each frequency was calculated as the ratio (multiplied by 100) between the peak height of the mutant nucleotide and the sum of the peak heights of the mutant and wild-type nucleotides: frequency = [mutant peak height / (mutant peak height + wild-type peak height)] × 100.
