# Supplementary material for: Navigating the Fitness Landscape: Host Density, Epistasis, and Clonal Interference Drive Divergent Evolutionary Pathways in Phage Qβ
Source: Int J Mol Sci. 2025 Sep 16;26(18):9020. doi: 10.3390/ijms26189020 (PMC12469967; doi:10.3390/ijms26189020)
Supplement: Supplementary file 1 [file ijms-26-09020-s001.zip › Figure S1.pdf]

Figure S1. AlphaFold 3 structural models of wild type (WT) and T222N, Q195R, and double T222N-Q195R Qβ A1 mutants colored by model confidence score pLDDT (box).

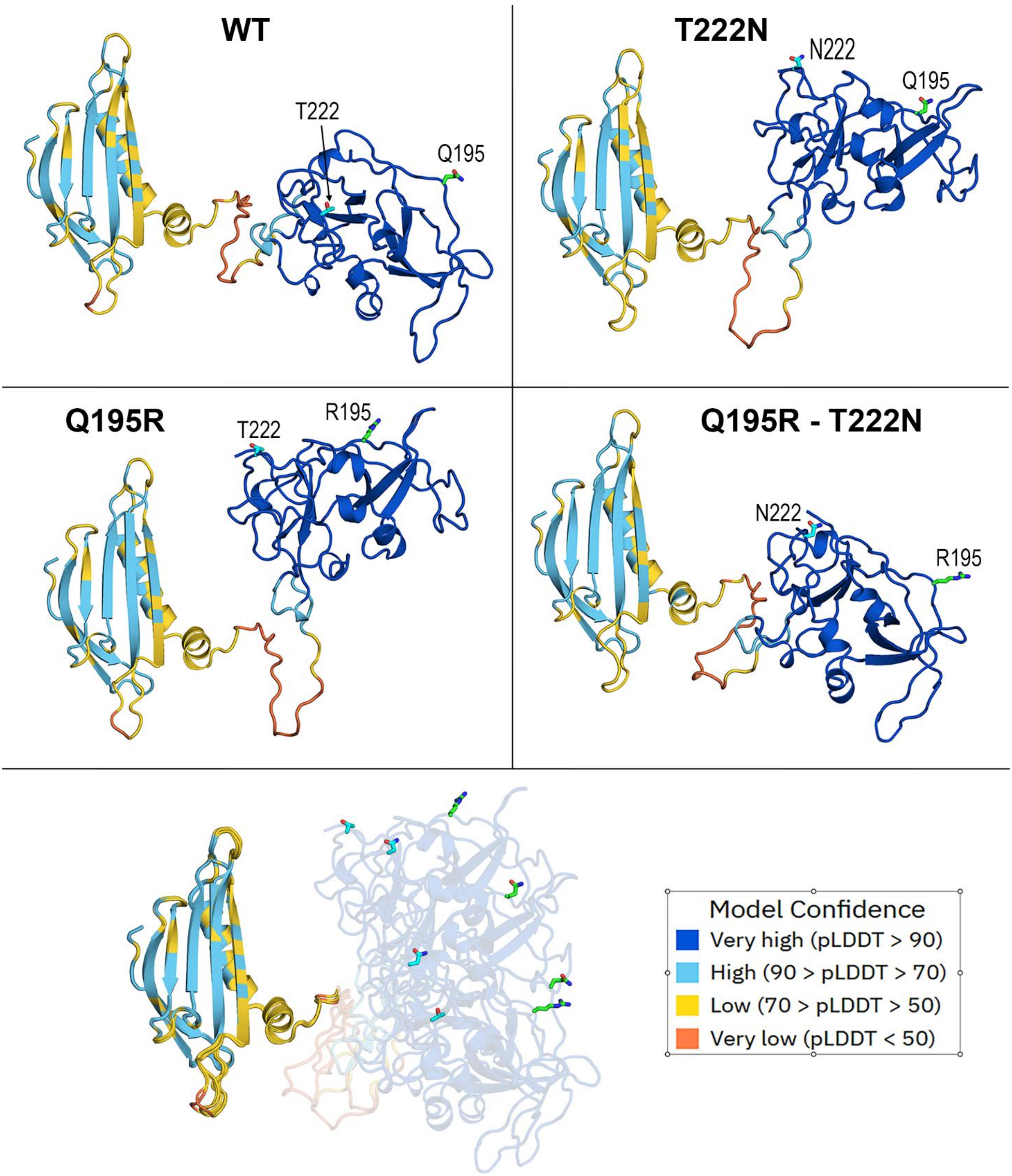

The four models are displayed at the orientation corresponding to their structural superposition on the CP domain shown below. Mutant residues are depicted as sticks with O atoms in red, N atoms in blue, and C atoms in cyan (222) or green (195). In the superposition, the A1 domain is

displayed at 80% transparency to highlight the position of residues 222 (cyan carbons) and 195 (green carbons).
